# Fake views removal and popularity on YouTube

**DOI:** 10.1038/s41598-024-63649-w

**Published:** 2024-07-04

**Authors:** Maria Castaldo, Paolo Frasca, Tommaso Venturini, Floriana Gargiulo

**Affiliations:** 1 Univ. Grenoble Alpes, CNRS, Inria, Grenoble INP, GIPSA-lab, F-38000 Grenoble, France; 2 CIS, CNRS, 59 Rue Pouchet, 75017 Paris, France; 3 University of Geneva, Geneva, Switzerland; 4 Gemass, CNRS, 59 Rue Pouchet, 75017 Paris, France

**Keywords:** Scientific data, Computational science, Applied mathematics

## Abstract

This paper analyses how YouTube authenticates engagement metrics and, more specifically, how the platform corrects view counts by removing “fake views” (i.e., views considered artificial or illegitimate by the platform). Working with one and a half years of data extracted from a thousand French YouTube channels, we show the massive extent of the corrections done by YouTube, which concern the large majority of the channels and over 78% of the videos in our corpus. Our analysis shows that corrections are not done continuously as videos collect new views, but instead occur in batches, generally around 5 p.m. every day. More significantly, most corrections occur relatively late in the life of the videos, after they have reached most of their audience, and the delay in correction is not independent of the final popularity of videos: videos corrected later in their life are more popular on average than those corrected earlier. We discuss the probable causes of this phenomenon and its possible negative consequences on content diffusion. By inflating view counts, fake views could make videos appear more popular than they are and unwarrantedly encourage their recommendation, thus potentially altering the public debate on the platform. This could have implications on the spread of online misinformation, but their in-depth exploration requires first-hand information on view corrections, which YouTube does not provide through its API. This paper presents a series of experimental techniques to work around this limitation, offering a practical contribution to the study of online attention cycles (as described in the “Data and methods” section). At the same time, this paper is also a call for greater transparency by YouTube and other online platforms about information with crucial implications for the quality of online debate.

## Introduction

“We want to make sure that videos are viewed by actual humans and not computer programs^1^.” According to multiple official web pages, YouTube applies sound checks to view counts and removes all the views that it deems done by automated programs. Consistently, the press has documented several major corrections events. In December 2012, for instance, the platform deleted 2 billion views from the channels of record companies such as Universal and Sony^2–5^ and, over the years, countless youtubers have suffered sudden and drastic cuts to their views (and many have complained about it, often through YouTube videos). According to YouTube’s policies^1,6,7^, these interventions aim at preserving a “meaningful human interaction on the platform” and to oppose “anything that artificially increases the number of views, likes, comments or other metric either through the use of automatic systems or by serving up videos to unsuspecting viewers^6^.”

Despite the media interest in the phenomenon^8,9^, researchers have not investigated in much detail the implementation and the effects of this policy. The lack of studies on the subject is partly motivated by the restrictions on data collection by YouTube’s API, which does not provide access to the number of removed views or to the evolution of the view count. The only information available through the API is the number of total views collected by a video until the moment of the query. Hence, studying the evolution of views (and of the removed views) requires reconstructing the time series through periodic queries to the API. The complexity of such a data collection is so daunting that, up to our knowledge, no work on view counts evolution has been done since 2017, when YouTube removed access to historical view counts evolution on its interface. Among the few studies on view counts evolution prior to 2017, only one dealt with view corrections, written by Marciel et al.^10^ in 2016. This paper studies the phenomenon of view corrections in relation to video monetization, in order to identify possible frauds, drawing on research on ad frauds in other social media^11,12^. In their work, Marciel et al. created some sample YouTube channels and inflated their views through bots, finding that “YouTube monetizes (almost) all the fake views” generated by the authors and “detects them more accurately when videos are not monetized”.

Other pressing questions, however, remain unanswered. In particular, we are interested in knowing whether fake views can influence the success of videos and be used to manipulate YouTube’s attention dynamics. It is well known that, on social media, future visibility is highly dependent on past popularity, as trending contents tend to be favored by human influencers^13^ and recommendation algorithms^14^, both of which are highly sensitive to metrics of trendiness^15^. On YouTube, in particular, the recommendation engine represents the most important source of views^16^ and, as admitted by its developers, “in addition to the first-order effect of simply recommending new videos that users want to watch, there is a critical secondary phenomenon of bootstrapping and propagating viral content^17^.” Therefore, YouTube’s algorithm creates positive feedback that skews visibility according to a rich-get-richer dynamic^18–20^: “models trained using data generated from the current system will be biased, causing a feedback loop effect. How to effectively and efficiently learn to reduce such biases is an open question^21^”. This is where fake views may come into play^22^.

Indeed, if the correction of illegitimate views happens too late, these views might weigh in the cycle of trendiness and unfairly propel their targets. If YouTube’s fake views correction is significantly slower than its recommendation dynamics, then artificially promoted videos risk being favored by human and algorithmic recommendations, thus reaching larger audiences and collecting extra real views. If, before being deleted, fake views can trigger a cascade effect that increases the visibility of some content, then they can be used to manipulate content diffusion. Similarly to social bots (fake accounts mimicking human behavior online)^23^, fake views could give the false impression that some content is highly popular and endorsed by many, thus fostering its further diffusion and manipulating the public debate^24–31^. While much research has been dedicated to social bots (to detect them^32–35^ and understand how they can enhance the diffusion of content^36–44^), more needs to be done on the artificial inflation of engagement metrics.

This work takes some first steps in understanding this pathological dynamics by studying how fake views are removed on YouTube. Our results highlight the risks associated with the delays in fakes views correction and suggests implications on the design of YouTube interface and recommendation system, which we present in the discussion. Our analysis shows how, on the platform, corrections do not occur continuously as new views are recorded, but are done in batches and at regular times during the day. This fact suggests that there can be delays between when fake views are made and when they are removed from the platform. Indeed, most corrections occur late in the life of videos, compared to when real views are collected. Moreover, videos with later corrections tend to become, on average, more popular than videos that are corrected earlier. A possible reason for this phenomenon is that fake views, when corrected too late, end up interfering with human and algorithmic recommendations and boost the spread of their targets.

Correlation, however, is not causation and, in order to build a definitive proof of the boosting effect of fake views on real views, we would need information that YouTube does not provide. In particular, we would need information about the exact moment in which fake views (or at least corrections) are made, as this information would allow us to study the temporal causality between fake and real views. As YouTube does not provide this information, our results remain unfortunately tentative and suggestive. Nevertheless, this work is a chance to discuss how greater access to platform data could improve the academic understanding of online content diffusion and the role that platforms play in shaping it, joining the calls for transparency made by other researchers^45,46^.

## Results

Starting in January 2021 and for 17 months, we monitored the view counts evolution of all the videos published by 1064 French YouTube channels dealing with news and politics. We recorded, in particular, the number of views collected *hour by hour* by 270,133 videos during the first week after their publication. As YouTube provides no direct information on its view corrections, the latter are estimated from the only retrievable information: the evolution of cumulative view counts. To do so, one possibility is to consider all the negative variations in the cumulative view counts as proxies for corrections: indeed, as users cannot *un*watch a video, if in 1 h we record less views than in the previous one, this means the platform intervened in removing some views. As we will discuss in the “Data and methods” section, this approximation heavily underestimates corrections due to the *hourly* sampling frequency.

To overcome this information loss, we collected a smaller dataset with higher frequency and used it to train a machine learning algorithm to reconstruct the real amount of corrections done by the platform. The results presented in this section refer to the dataset reconstructed through this machine learning algorithm, which is described in the “Data and methods” section.

**Figure 1:**
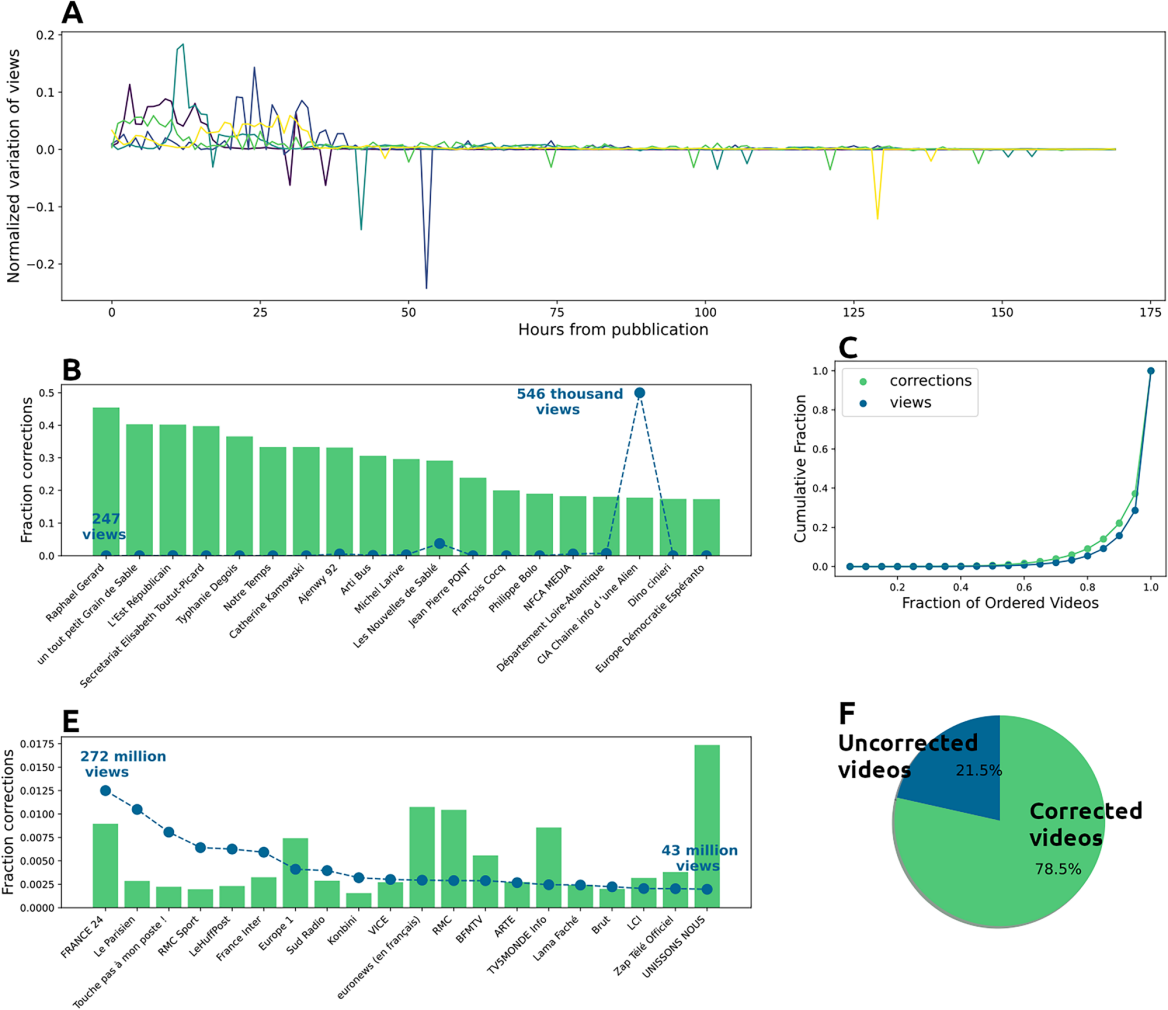
(**A**) 5 sample videos and their hourly evolution of views. (**B**) Fraction of corrections over real views (histogram) and number of views (blue line) for the 20 most corrected channels in terms of fraction of corrections. (**C**) Fraction of corrections over real views (histogram) and number of views (blue line) for the 20 most viewed channels in the dataset. (**D**) Lorenz curve of the distribution of views and corrections among different videos. (**E**) Percentage of videos affected by corrections.

### Scale of the phenomenon

The removal of fake views is evident when the series of hourly views has negative entries: some examples are shown in Fig. 1A. This phenomenon, we found, is surprisingly common. We detected corrections for almost all monitored channels (90% of them) and for 78.5% of the videos in our corpus (Fig. 1E). Corrections in our corpus amount to about 22.5 millions. Although they only represent 0.5% of the total views, their number remains impressive and, more importantly, their distribution is very uneven. If we look at the Lorenz curve (Fig. 1C) of the distribution of corrections among different videos, we can see that most of the corrections (more than 80%) are concentrated on only 20% of the videos. The heterogeneity of the corrections is confirmed when we examine the most popular and the most corrected videos. Figure 1D shows the 20 most popular channels in our dataset and the percentage of actual views that corrections account for. These very popular channels—mainly traditional media channels such as TV stations, newspapers, and radio stations—show marked differences in their corrections (between 0.1 and 0.17%). Looking at the 20 channels with the highest fraction of corrections to actual views (Fig. 1B), we find channels with 40% corrections. These channels are mostly platform-native youtubers, collecting far fewer views than the top channels.

### Correction rhythms

**Figure 2:**
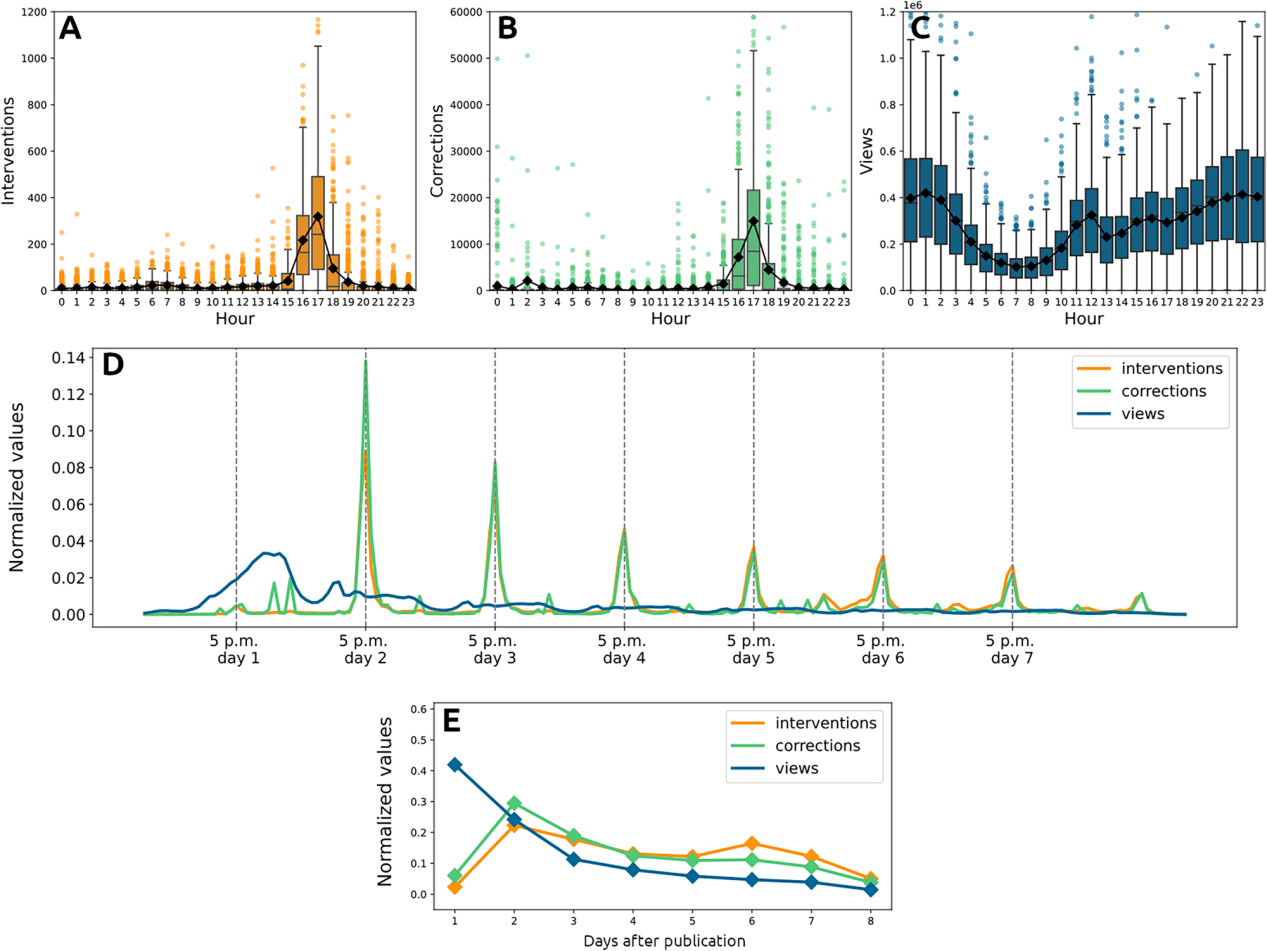
(**A**) Distribution of interventions per hour of the day. (**B**) Distribution of corrections per hour of the day. (**C**) Distribution of views per hour of the day. (**D**) Normalized number of interventions, corrections and views per hour following publication. (**E**) Normalized number of interventions, corrections and views per day after publication.

The correction activities by YouTube exhibit interesting recurring patterns and daily rhythms. Figure 2B, shows the daily distribution of fake views corrections (i.e., the number of removed views). While the mean number of removed views hovers around a few dozen at most hours, it leaps to about 9000 at 4 p.m. and to more than 16,000 at 5 p.m. Similarly, looking at the number of *interventions* (Fig. 2A) (i.e., the events in which the platform removes some views, regardless of the amount removed), we observe that, while the mean number of is generally close to zero, between 4 p.m. and 5 p.m. it spikes up to more than 250. These rhythms of correction are peculiar and completely different from the rhythms at which views are made on the platform. Figure 2C shows how the daily distribution of views reaches its minimum in the morning around 7 a.m. and peaks in the late evening (9 p.m. to 2 a.m.). Hence, while views are distributed quite evenly during the day, following well-documented circadian rhythms^47^, corrections are concentrated at specific times. The periodicity of corrections and interventions becomes even more evident if we look at how they are distributed during the life of videos (Fig. 2D). This evidence indicates that corrections are performed in batches rather than continuously as new views are recorded. These corrections occur sporadically, perhaps once a day, following a schedule unrelated to viewing patterns. They are relatively infrequent when compared to the rapid propagation of content on the platform and the regular updates made by recommendation systems. As a point of comparison, the majority of videos in YouTube’s “trending” section (https://www.youtube.com/feed/trending) are less than 24 h old. In fact, after analyzing the top 20 videos in the trendy section on seven different days, we discovered that only 25% had been published for more than a day. This raises the question of whether such a low correction frequency is adequate in preventing fake views from influencing both human and algorithmic recommendations. Another interesting evidence comes from the aggregation by day of the distribution of views, corrections and interventions (Fig. 2E). As we can see form the plot, views are mainly concentrated on the first day of videos’ lives and decrease afterwards. On the contrary, corrections are mainly concentrated on the second day and then they decrease and interventions present two peaks of activity, one on the second day and one on the sixth, suggesting the presence of further checks in the later life of videos.

### Late corrections and popularity

**Figure 3:**
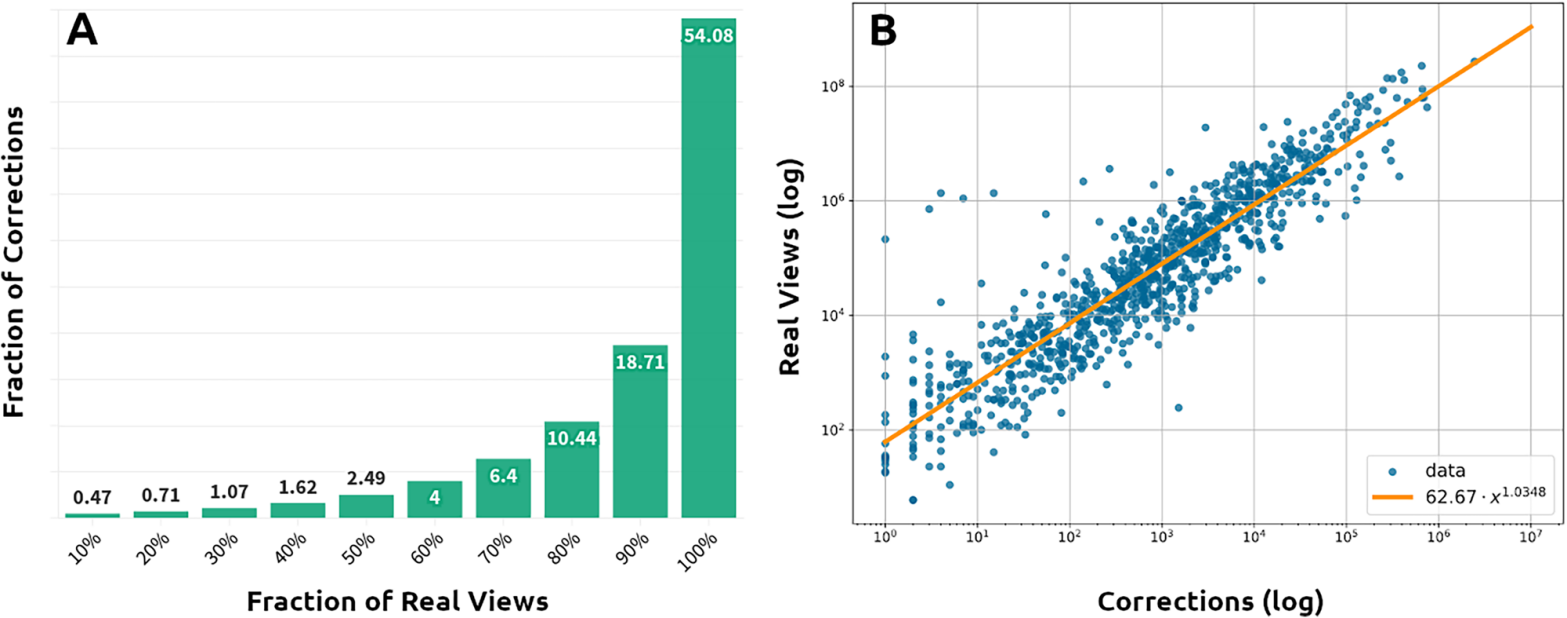
(**A**) Fraction of corrections occurring after different percentages of real views. (**B**) Correlation between real and fake views per channel.

Besides looking at the absolute timing of corrections, we would like to investigate when corrections are done in comparison to the attention cycle of a video and whether corrections are done before or after a video reaches its peak of popularity. If corrections occur after this peak, one might suspect that by inflating the number of views, illegitimate views could make a video appear more popular and unfairly boost its human and algorithmic recommendation. Figure 3(left) presents the percentage of corrections made before videos reach a given percentage of their total real views. As the graph shows, most of the illegitimate views are corrected *after* the videos have garnered most of their real views. On average, only about 10% of the corrections are made before the videos have achieved 80% of their views. Even more striking is the fact that as many as 54% of corrections are made after the videos have stopped collecting any views at all, at the end of their lives. It is therefore quite clear that most illegitimate views are removed very late in the lives of videos, well after their popularity has peaked and has begun to decline.

To understand whether this delay has an effect on the success of videos, we investigated the relationship between fake views and popularity. Figure 3(right) examines the relationship between the number of fake views and the total number of legitimate views per channel. In principle, these two quantities should be independent: in fact, if YouTube’s corrections were immediate and perfectly effective, illegitimate views should have no impact on real views.

**Figure 4:**
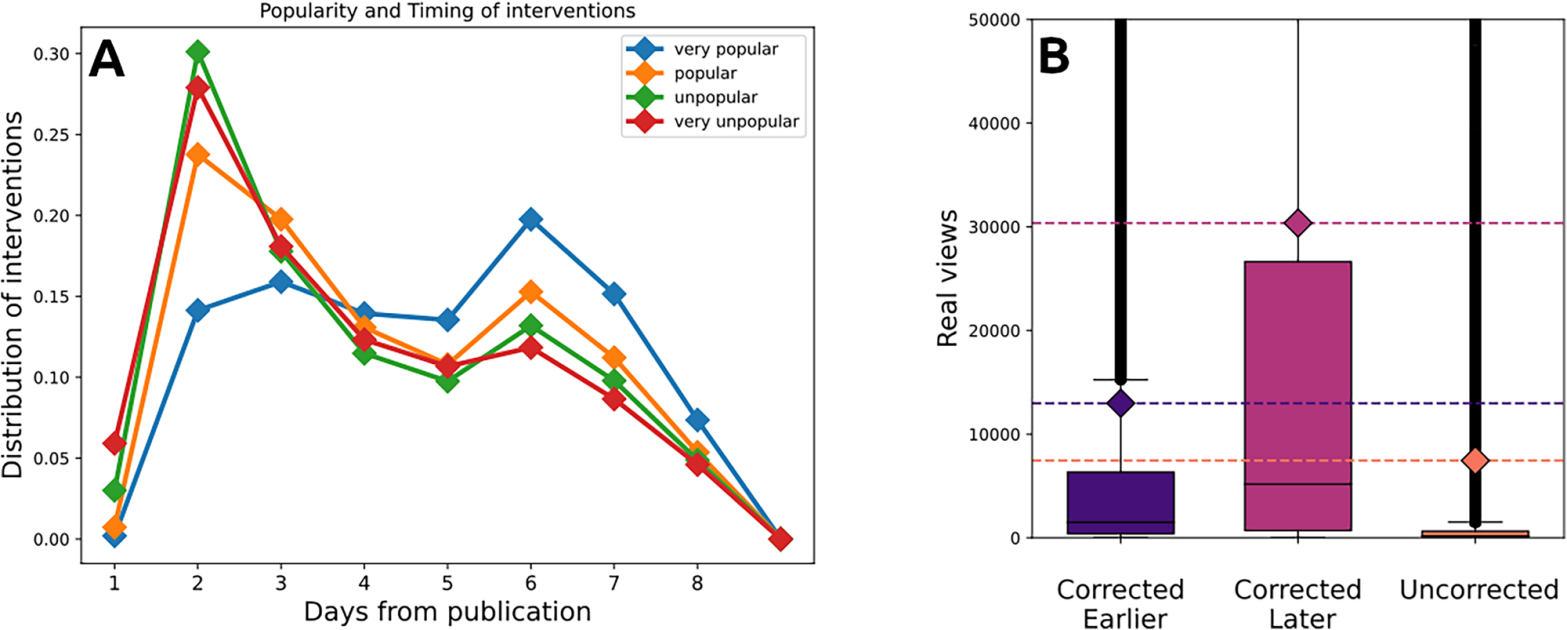
(**A**) Distribution of interventions in the days following the publication. (**B**) Distribution of popularity for videos corrected earlier, later and uncorrected.

Yet, Fig. 3(right) shows a strong linear correlation between the logarithms of the two quantities. With a R-square equal to 0.826, the logarithms of fake and real views are related by a linear regression with intercept equal to 1.7971 and slope equal to 1.0348. The p-values associated with these quantities are very low, less than 0.0001. This linear correlation between the logarithms of the two quantities results in the following relationship between corrections (*c*) and real views (*v*):

$$\begin{aligned} c = 62.67 \cdot v ^{1.0348}. \end{aligned}$$

The relationship between these two quantities is hence slightly more than linear, with a $95\%$ confidence interval on the exponent being within $[1.005, \; 1.065]$.

This indeed indicates that fake and real views are not independent variables. This fact, together with the fact that the majority of the corrections happens *after* the videos have collected most of their real views, suggest that due to the late correction, fake views could affect the final popularity of videos. However, relying solely on linear regression to understand the relationship between real and fake views has its limitations. Notably, correlation does not imply causality, and in this context, linear regression encounters an endogeneity problem^48^. Endogeneity arises from the possibility that the explanatory variable (fake views) may be influenced by the dependent variable (real views), or both could be jointly influenced by an unmeasured third variable. Such endogeneity has the potential to introduce bias into our analysis.

Various strategies exist in literature to address endogeneity issues, but their application in this context proves challenging. For example, instrumental variables (IV) analysis^49^ necessitates the identification of a third variable correlated with fake views but independent of real views. Unfortunately, the data available through the platform only provide access to engagement metrics (such as the number of likes, comments, and subscriptions) and information about the channel type. However, engagement metrics are inherently correlated with views, rendering them unsuitable as instrumental variables. Similarly, while the type of channel could potentially serve as a candidate instrumental variable, our observations indicate that the phenomenon of fake views affects virtually all channels in our corpus (90%), suggesting that channel type would not be a good choice as an instrumental variable either.

Another approach involves causality tests based on temporal analysis, aiming to establish the temporal sequence of events for inferring causality. However, while we can track the evolution of the number of views over time, there is no way to accurately distinguishing between when fake and real views are recorded. As a result, determining their temporal order becomes impractical. The only temporal evidence we have is the moment when fake views are removed. By examining the timing of view removals in correlation with video popularity, we aim to shed light on the dynamics between real and fake views, bypassing the limitations associated with endogeneity and circumventing the constraints of data gathering imposed by the platform. To this end, we divide the videos in our dataset into four classes of popularity: *very popular/popular/unpopular/very unpopular* according to the four quartiles of the distribution of final views. Figure 4A presents, for each class, the normalized number of YouTube interventions by day. The figure shows that the lowest popularity quartiles are corrected earlier, mainly on the second day after publication, while the most popular quartile is mainly corrected on the 6th day following publication. Hence, more popular videos tend to be corrected later than less popular ones. We could hence wonder if, conversely, videos corrected later are more popular. To answer this question we divided videos into three classes: videos *corrected earlier* (presenting the majority of the interventions before the 4th days), videos *corrected later* (presenting the majority of the interventions after the 4th day), and *uncorrected* videos (with no intervention). Figure 4B shows the distribution of final views for these three classes. As we can see, videos corrected later are on average more popular than videos corrected earlier and than uncorrected videos. The difference between the distributions of popularity of the three classes of videos has been tested through a two-sample Kolmogorov Smirnov test and all p-values were found to be less than $10^{-5}$. Videos corrected later present an expected popularity 3 times greater than videos corrected earlier and almost 4 times greater than uncorrected videos. Hence we can say that videos corrected later in general reach wider audiences.

## Discussion

In summary, our analysis has led to four main findings. Our first finding concerns the remarkable size of the corrections phenomenon, which involves almost all the monitored channels and 78% of the videos. Our second finding is that the rhythms of view corrections are inconsistent with those of view production, suggesting that they happen in batches and not continuously as new views are recorded. Moreover, they happen late in the life of videos, as most of them (almost 90%) occur after videos have already reached 80% of their final audience. Our third finding is a correlation between corrections and real views: videos with more corrections also collect more legitimate views, and the other way around. The fourth finding deals with the relationship between correction speed and final popularity. Videos on which the platform intervenes later are, on average, more popular than those corrected earlier.

An explanation of these results could come from a possible interference of fake views with the dynamics of content diffusion on the platform. Fake views could give a false impression that a video is highly popular and, in this way, boost its further diffusion through human and algorithmic recommendation. Indeed, rich-get-richer dynamics have repeatedly been observed in the platform’s evolution of view counts^50^. Earlier works have often credited the total number of previous views as the most important predictor of future popularity^19–50^. Moreover, rewarding trendy content with more visibility is a central feature of the recommendation system, which–according to YouTube developers–aims at “bootstrapping and propagating viral content”^17^. While encouraging the diffusion of trending videos, the recommendation system is also the primary source of views for most YouTube videos^16,51^. Recommendations are updated relatively quickly: according to Roth et al., for instance, two-thirds of the suggestions are associated with a video for less than 2 days^52^. Such a fast refreshing of suggested videos, along with the massive impact of the recommendation system, warrants concerns about view correction being done in batches and late in the life of videos. Moreover, these popularity dynamics could explain why videos corrected later are, on average, more popular than those corrected earlier. With a delay in correction, videos could have enough time to be recognized as viral and thus reach a wider audience than they would have reached without illegitimate boosting.

Deepening the understanding of this phenomenon, however, would require a crucial piece of information not available or derivable from the data published by YouTube: the time at which fake views are made. Not only is this information not provided by the API, but there is also no way to estimate it through the view counts evolution. Knowing when fake views are made would allow us to assess the temporal order in which videos collect fake and real views and investigate the temporal causality between these two phenomena. In the absence of this information, we can only base our analysis on the time at which they are removed, which itself needs to be estimated as we have done in this work. Despite these limitations, our results robustly indicate the massive scale of views’ corrections and suggest the existence of a positive association between the illicit boosting and the popularity of YouTube videos. Our results highlight the crucial role of time in the diffusion of online content and reveal the role that even a relatively short delay in the correction of views frauds may play in visibility trends.

Further research would now be required to investigate whether the videos or channels that accumulated most fake views are also those that are more likely to spread misinformation, click-baits, sensationalist content, or have in other ways a similar features in term of the videos they publish or the profile of their followers and commentators. This investigation, however, would require an even larger corpus than the one we collected, which for reasons explained in the “Data and methods” section is difficult to obtain.

Given the importance of the subject and the potential harm from the malfunctioning of the correction policy, our findings should therefore encourage YouTube to include in its API the number of corrected views for each video. As we have shown, this information is crucial to investigate the alarming possibility that relatively easy techniques of views inflation, if used early enough in the life of a video, could effectively manipulate the visibility by triggering the trending dynamics sustained by manual and algorithmic recommendation. As other researchers have claimed in the last few years^45,46^, we believe that a more comprehensive access to platform data is crucial to study the possible malfunctioning of its moderation policies. In particular, access to removed content (like removed comments or views in our case) would help investigate the effects they triggered before the takedown and study, for instance, their potential role in spreading misinformation^46^.

If confirmed, the risks of visibility manipulation highlighted by our investigation advocate for some rethinking of YouTube recommendation system and graphical interface. Indeed, should the platform prove incapable to correct fake views more timely, it might consider slowing down its recommendation algorithm to match the speed of the illegitimate views detection, as well as hiding, or at least preemptively reducing, the number of views displayed in the interface for the first few hours after the publication of each video. This will give YouTube time to carefully assess which contents deserves to be pushed to and by its audience. We understand that, in the extremely competitive attention economy in which the platform operates, this might come at a cost to the platform. Yet avoiding the manipulation of online debate is crucial for our societies, and all platforms should put in place all possible guardrails to assure the health of the debates that they mediate.

## Data and methods

### Data

As suggested above, the contribution of our work lies as much in our methods and in our data as in our results. The problem of studying attention cycles in online platforms is not limited to YouTube, and neither is the problem of having to deal with APIs that do not time-stamp visibility metrics such as views, likes and shares, but only return their total at the *time they are queried*. This lack of historicity is a problem for all researchers who want to know, not only how popular a piece of content or a topic is, but when and how quickly it reached its peak of popularity. In this section, we therefore detail our struggle with YouTube’s lack of transparency and the sometimes unconventional solution we had to find, in the belief that this can help other scholars carry out similar research.

For our analysis, we used a large dataset sampled with hourly frequency, which we will refer to as the *hourly dataset*. We also collected a smaller dataset, sampled with a 5 minute frequency, which we refer to as the *5 minute dataset*: its main purpose is to assess how much information is lost through the hourly sampling and to test our methods to reconstruct the lost information. The details of the two datasets are presented next.

#### Hourly dataset

Starting from January 2021, through a collaboration with the Qatar Computing Research Institute (QCRI), we collected the time evolution of views of all the videos published by a list of over a thousand French YouTube channels dealing with news and politics. This dataset is particularly interesting as it cannot be retrieved *a posteriori* through the official YouTube application programming interface (API)^53^. Indeed YouTube’s API only returns the number of views a videos has in the moment of the query and it does not provide the historical evolution of view counts. The only way to collect view counts evolution is through constantly querying the API over the period of interest. Hence, without querying the API every hour for the last year and a half, the information collected in our dataset would have been completely lost (for researchers) and in no way retrievable.

Our dataset covers 1064 popular French channels that, in discussion with experts, were recognized as particularly influential in the French public debate. These channels, with their description, are available on Figshare^54^. The channels have been selected through a qualitative analysis of the French YouTube, aiming to identify relevant actors that diffuse political opinions through the platform. The selected channels belong to the following categories: local and national media; influential YouTubers discussing political topics; militant associations; politicians; political parties; Yellow Vests groups; associations devoted to public causes; large public or private institutions. YouTube provides no information about the location from which videos are viewed, but since the channels of our corpus focus on French public debate, we can assume most of their viewers to be based in France.

For these channels, we recorded hour by hour the evolution of views of each video published after the 1st of January 2021, for an entire week after publication (170 hours). Between the 1st of January 2021 and the 10 of May 2022, we collected the views time series of 270.133 videos. The dataset is available on Figshare^55^ (resource IDs have been mapped to anonymize values consistently along the dataset). The choice of collecting only 1 week of views evolution is justified by noticing that news channels often collect the majority of their views in few days after publication, presenting a strong initial burst followed by a power-law decay^56^. Indeed our data confirm this fast decrease of users engagement as only $3\%$ of views is obtained in the last 24 h. The choice to collect views every hour was dictated by the constraints on YouTube’s API, which does not allow more than 10,000 requests per day with the same access key. As a result, wanting to monitor the French news and political sphere on YouTube, and hence a certain amount of videos every day, we were forced to collect the data with this frequency.

#### 5 minute dataset

To assess the amount of information lost by collecting data every hour, we collected a smaller dataset with 5 minutes frequency. This frequency has been chosen so to minimize the information loss and is in practice the highest useful frequency of data collection, since we have empirically observed that view counts are updated by the platform no faster than every 5 minutes. This dataset contains views time series of 1934 videos posted by our channels of interest between February 2, 2022 and February 16, 2022. The dataset is available at Figshare^57^. Video identifiers have been anonymized consistently with the hourly dataset.

### Methods to estimate corrections

To reduce the loss of information due to collecting data on an hourly basis, we have devised a method to reconstruct the original corrections and interventions. To remove biases caused by the hourly sampling, we need to infer the *real* corrections $c_h^i$ made by the platform at hour *h* on video *i* from the observable quantity ${\tilde{v}}_h^i = v_h^i - c_h^i$, where $v_h^i$ are the effective non-observable views collected by video *i* during hour *h*, and $c_h^i$ are the effective corrections made by the platform. In order to quantify the performance of our reconstruction methods we use the corrections and interventions visible in the 5-minute dataset as the best proxy for the real corrections $c_h^i$. In general, we would like the estimated values ${\hat{c}}_h^i$ to reduce the following errors:

- the fraction of *lost corrections*, consisting of the fraction of real corrections that are lost in the reconstructed series;
  $$\begin{aligned} \frac{\sum _{h,i: c_h^i > {\hat{c}}_h^i} (c_h^i - {\hat{c}}_h^i) }{\sum _{h,i} (c_h^i)} \end{aligned}$$
- the fraction the *added corrections*, consisting of the corrections mis-added by the reconstruction methods, divided by the total real corrections;
  $$\begin{aligned} \frac{\sum _{h,i: {\hat{c}}_h^i > c_h^i} ({\hat{c}}_h^i - c_h^i) }{\sum _{h,i} (c_h^i)} \end{aligned}$$
- the fraction of *lost interventions*, consisting of the fraction of interventions no longer visible in reconstructed series;
  $$\begin{aligned} \frac{\sum _{h,i: c_h^i = 0 } \mathbbm {1}_{ {\hat{c}}_h^i>0 }}{\sum _{h,i} \mathbbm {1}_{ c_h^i> 0}} \end{aligned}$$
- the fraction of *added interventions*, consisting of the number of mis-added interventions by reconstruction methods, divided by the total number of real interventions.
  $$\begin{aligned} \frac{\sum _{h,i: \hat{c}_h^i = 0 } \mathbbm {1}_{ c_h^i>0 }}{\sum _{h,i} \mathbbm {1}_{ c_h^i> 0}} \end{aligned}$$

When approximating the corrections with the negative views visible in the hourly collection, i.e., by taking $c_h^i = -{\tilde{v}}_h^i \mathbbm {1}_{\{v_h^i>0\}}$, we obtain the errors shown in Table 1. As the table shows, the loss of information is far from being negligible, since we loose 66.31% of the corrections and 60% of the interventions.

**Figure 5:**
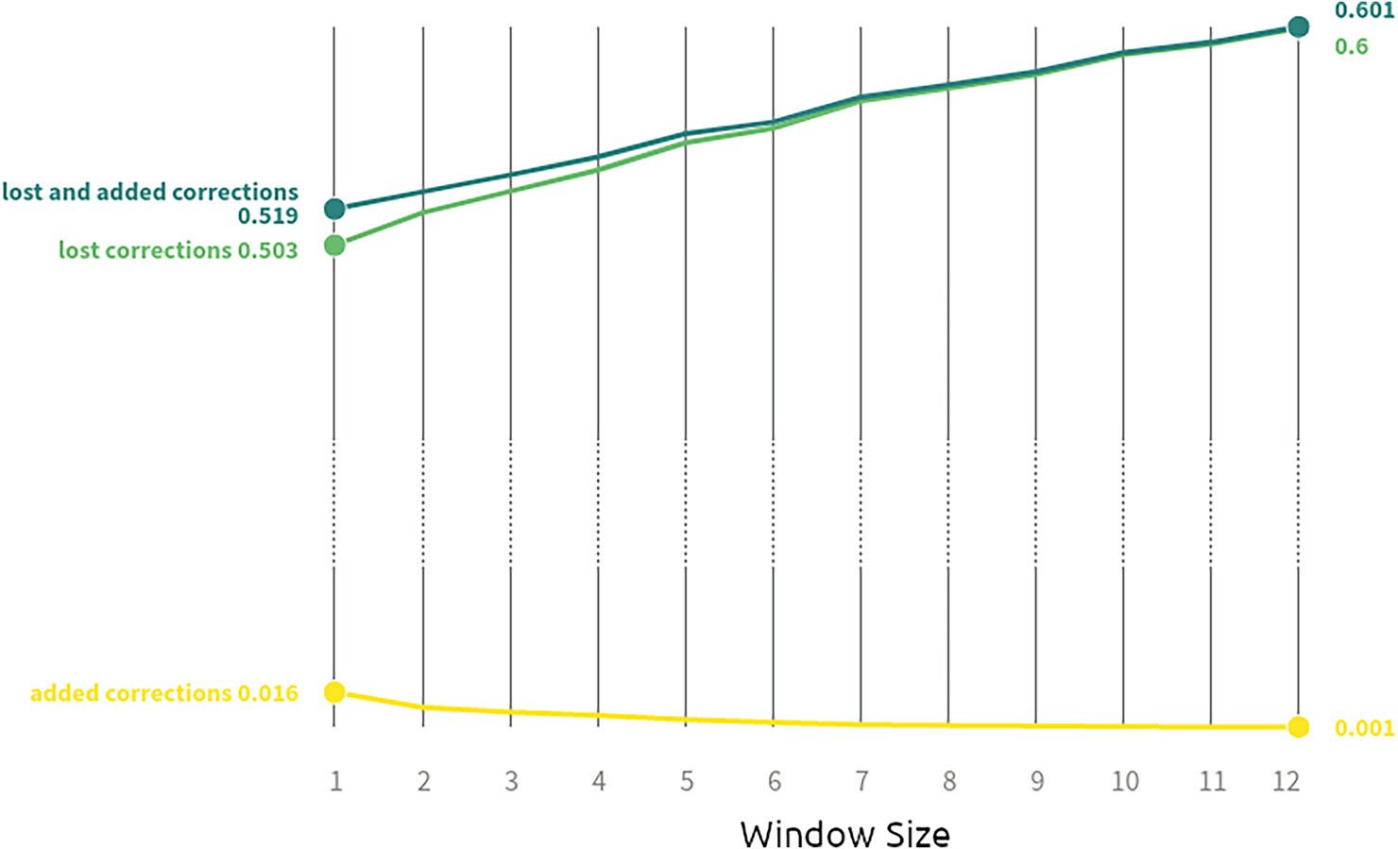
Error introduced by the benchmark method, varying the time window.

**Figure 6:**
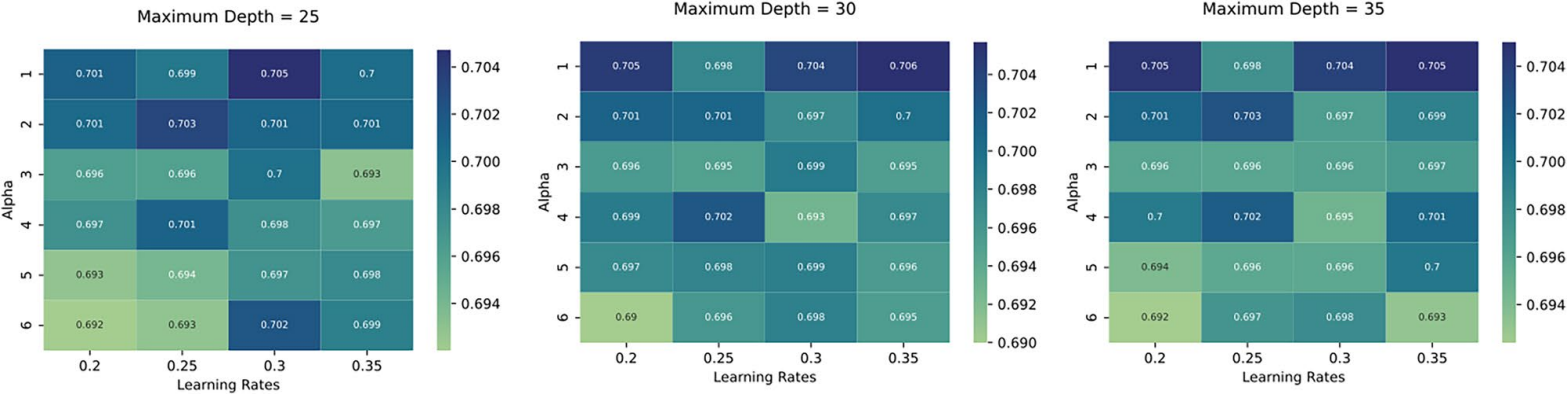
XGBoost parameter tuning. Performances in terms of F1 score associated with different combinations of parameters’ values.

In the following we present two methods to reduce this information loss. The first, which we will refer to as the *benchmark method*, is simpler and uses a heuristic. The second method, which we will refer to as the *reconstruction method*, uses an XGBoost classifier^58^ to improve the benchmark method and effectively reduce information loss in the reconstructed series.

**Table 1:** Validation of reconstruction method on the 5-minute-frequency dataset.

	Hourly aggregation (%)	Benchmark method (%)	Reconstruction method (%)
Lost corrections	55.66	42.31	31.06
Added corrections	0	1.25	8.97
Lost interventions	61.12	61.12	29.31
Added interventions	0	0	4.06

The table shows the loss of information with hourly aggregation and with the estimates done with the proposed Reconstruction Method and the Benchmark method.

#### Benchmark method

As a benchmark method we propose the following heuristic: in hours with negative views, corrections are approximated by the number of negative views increased by the expected views at that hour. To estimate the expected views in a certain hour *h* we use a function *f* of the views in the *k* hours preceding and following hour *h*:

1 $$\begin{aligned} {\hat{c}}_h^i = (-v_{h}^i + f (v_{h-k}^i,\ldots ,v_{h-1}^i, v_{h+1}^i, \ldots ,v_{h+k}^i)) \mathbbm {1}_{\{v_h^i>0\}} \end{aligned}$$

As for the function *f*, we tested the performances of different functions: the minimum, the mean, the maximum. We did the same for different values of the time window’s size *k*. For instance, Fig. 5 shows the errors associated to the choice of *f* as the *min* and different window’s sizes. The best choice to minimize the sum of lost and added corrections was to take *f* equal to the minimum over a 1-h time window. Hence we approximate:

2 $$\begin{aligned} {\hat{c}}_h^i = (-v_{h}^i + \min (v_{h-1}^i, v_{h+1}^i )) \mathbbm {1}_{\{v_h^i>0\}} \end{aligned}$$

This benchmark method reduces the lost corrections from 55.66% to 42.31% by introducing only the 1.25% of added corrections.

#### Reconstruction method

The above benchmark method adjusts negative views to estimate the platform corrections, but is unable to detect correction events that have occurred when the observed views ${\hat{v}}_h^i$ are nonnegative. Hence we developed a method meant to detect anomalies in the views evolution and attribute them to concealed corrections. The method consists of an XGBoost classifier that can detect hours with unobserved corrections. Below we present how this classifier was trained and we analyze its performance.

We constructed the train dataset as follows:

- each row represents 1 h of our time-series;
- for each hour we extracted the evolution of views in the 12 h before and after that hour and added them as features;
- we added as features the time of day (since corrections are roughly periodic and occur mostly between 4 and 6 p.m.) and the number of hours and days elapsed since publication.

In order to do parameter tuning, we chose to optimize the F1 score, a metric defined as follows:

$$\begin{aligned} \text {F1} = \frac{2}{\frac{1}{\text {precision}}\times \frac{1}{\text {recall}}} \end{aligned}$$

where *precision* stands for the rate of true positives over all the samples classified as positive, while *recall* stands for the rate of true positives over all the really positive samples in the data. In this way we can limit the number of false positives and false negatives introduced by our classifier. We divided our dataset into a train set of 90075 observations and a test set of 24674 observations. We used the train set to perform a 5-fold cross validation to choose the optimal values of *maximum depth* of the decision trees, the *learning rate* and *alpha* parameters of the XGBoost classifier. The performances in terms of F1 score associated with different combinations of values are shown in Fig. 6. The best parameters, able to grant an F1 score equal to 0.706, are maximum depth equal to 30, learning rate equal to 0.35 and alpha equal to 1.

The classifier allows us to reconstruct which hours have YouTube interventions without the latter being visible in the hourly aggregation. Once we identify the hours with interventions, we estimate the magnitude of the corrections by formula (1). In this way, as shown in Table 1, we are able to reduce the lost interventions from 61.12% to only 29.31% and the lost corrections from 55.66 to 29.31%.

#### Robustness of the results

**Figure 7:**
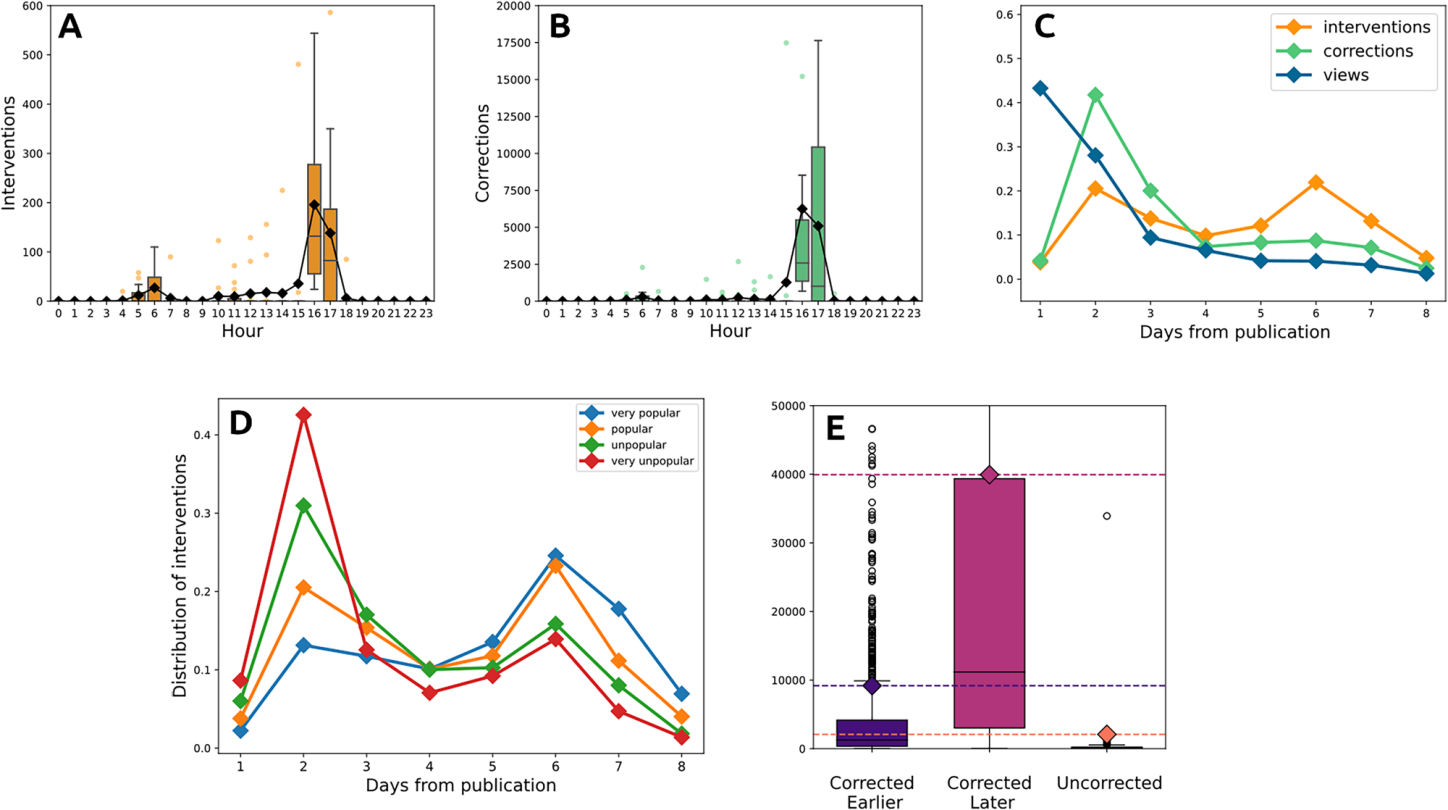
Consistency with 5-min dataset. (**A**–**C**) Analysis on the distribution of intervention and corrections. (**D**,**E**) Analysis on the relation between speed of correction and popularity.

To verify that our results are not artifacts introduced by our reconstruction method or caused by information loss due to the sampling frequency, we repeated our analysis on the 5-minute dataset. The results are similar to the ones of the hourly dataset, proving the robusteness of our findings. For instance, in Fig. 7 we can observe how the corrections and interventions remain periodic, with most of the corrections occurring between 4 and 5 p.m., just as shown in Fig. 2. We can also observe how interventions rise on the sixth day following the publication. As for the analysis of the relationship between correction speed and popularity, the results presented in Fig. 4 are confirmed. Videos corrected later are, on average, more popular than those corrected earlier or uncorrected. The fact that the 5-minute dataset confirms our results allows us to rule out the possibility that what we observe in the reconstructed series is an artifact due to the sampling rate or the methods used to reconstruct the time series.

## Data Availability

The datasets analysed during the current study are available on Figshare. In particular: the channel list is available at https://doi.org/10.6084/m9.figshare.20079584, the hourly dataset described in the “Data and methods” section is available at https://doi.org/10.6084/m9.figshare.20079857.v2, the 5-minutes frequency dataset described in the Data and Methods Section is available at https://doi.org/10.6084/m9.figshare.20080019.v5. Data is available on Figshare^54,55,57^.
